# Evidence for the Treatment of Osteoporosis with Vitamin D in Residential Care and in the Community Dwelling Elderly

**DOI:** 10.1155/2013/463589

**Published:** 2013-08-24

**Authors:** John A. A. Geddes, Charles A. Inderjeeth

**Affiliations:** ^1^Older Persons Health Specialist Service, Canterbury District Health Board, Christchurch 8022, New Zealand; ^2^Department of Medicine, University of Otago, Christchurch 8140, New Zealand; ^3^North Metropolitan Area Health Service, Perth, WA, Australia; ^4^University of Western Australia, Perth, WA, Australia; ^5^Sir Charles Gairdner Hospital, Hospital Avenue, Nedlands, WA 6009, Australia

## Abstract

*Introduction*. Vitamin D is common treatment for osteoporosis. Both age >70 years and living in residential care are associated with increased fracture risk. Community dwelling elderly are a heterogeneous group who may have more similatiry with residential care groups than younger community dwelling counterparts. *Aims*. To review the evidence for cholecalciferol or ergocalciferol tretment of osteoporosis in either community dwelling patients aged ≥70 years of age, or redidential care patients. Secondly endpoints were changes in bone mineral denisty, and in bone turnover markers. *Methods*. We performed a literature search using search terms for osteoporosis and vitamin D. Treatment for at least one year was required. *Results*. Only one residential care study using cholecalciferol, showed non-vertebral and hip fracture reduction in vitamin D deficient subjects. In the community setting one quasi randomised study using ergocalciferol showed reduction in total but not hip or non-vertebral fracture, and a second randomised study showed increased hip fracture risk. Three studies reported increases in hip bone mineral denisty. *Discussion*. A minority of studies demonstrated a fracture benefit form vitamin D and one suggested possible harm in a community setting. Current practice should be to only offer this treatment to subjects identified as deficient.

## 1. Introduction

Osteoporosis is common in the elderly [1]. The associated burden of minimal trauma fracture is expected to increase as populations age [1, 2]. Both age more than 70 years and living in residential care are associated with osteoporosis and increased fracture risk [3, 4]. The prevalence of osteoporosis in individuals aged more than 70 years has been shown to be 40% at the lumbar spine and 24% at the hip [4]. Age more than 70 years is associated with fractures in high-risk individuals [3]. Osteoporosis has been reported in 86% of nursing home residents [5], and the rate of fracture in individuals in residential care is 2.5–10 times higher than that in the community [6].

Falls are strongly implicated in the aetiology of fractures in the elderly [7, 8]. A third of all women aged more than 70 fall each year [9]. The rate of falling in residential care is higher than that in the community setting [10], which may have a role in the increased fracture rate of that environment.

A process of decreased osteoclastic and osteoblastic activities is associated with aging, contributes to osteoporosis in both men and women, and affects mainly cortical bone. Vitamin D induces osteoblastic activity, and a deficient state, as is common in older people, may contribute further to the development of osteoporosis with increasing age. In contrast, oestrogen deficiency, as is seen in the postmenopausal state, affects mainly trabecular bone and is primarily due to changes in osteoclastic activity [11]. In addition, vitamin D increases intestinal absorption of dietary calcium, aiding calcium balance and parathyroid hormone regulation, and also improves proximal muscle function, particularly when treating deficiency states [12]. Hypovitaminosis D is common in the elderly [12, 13], particularly in those sustaining hip fracture [14] or living in residential care [10]. Hence, in an aging population, the use of vitamin D would aim to prevent fracture through improved skeletal health and calcium absorption and by reducing antecedent falls. 

## 2. Objectives of This Review

To review the published literature on the clinical efficacy and safety of vitamin D in the form of ergocalciferol and cholecalciferol in the reduction in fracture risk in the frail elderly, the following major endpoints were used:

1. minimal trauma fracture reduction;
2. nonvertebral fracture;
3. hip fracture reduction;
4. BMD changes;
5. biochemical changes.

## 3. Search Methods for Identification of Studies

An electronic search of Medline (1970 to June 2010), EMBASE (1970 to June 2010) and the Cochrane Library (1996 to June 2010), using search terms for osteoporosis and vitamin D was performed. We reviewed the reference lists of identified articles for additional relevant published trials.

## 4. Eligibility Criteria for Inclusion in Review

Studies were randomised placebo or comparator control trials of at least 1-year duration and included both males and females. For eligibility studies were either of residential care populations or used age ≥70 as an entry criteria. Published subgroup analyses, specifying subgroups aged ≥70 year, were acceptable. Further inclusion criteria included use of precursor forms of vitamin D either as ergocalciferol (vitamin D2) or cholecalciferol (vitamin D3) and the reporting of fracture or bone mineral density (BMD) as primary outcomes.

Studies were excluded if they used active metabolite forms of vitamin D, had no control or placebo group, or if they were pooled analysis such as meta-analysis.

Abstracts of all possibly relevant articles were reviewed for potential eligibility by a single reviewer (John A. A. Geddes). Those records deemed eligible and those without adequate information to confirm their inclusion underwent full text review.

All data was summarised in a pre-formulated proforma including inclusion criteria, gender, age, type of study, duration and the main outcome measures. A second reviewer (Charles A. Inderjeeth) reviewed all publications meeting the inclusion criteria as well as those deemed to meet some criteria and helped resolve difference in interpretation with the initial reviewer (JG). 

All studies and subgroup analysis specifically reporting outcomes according to the prespecified criteria were included in the final review. No further or separate subgroup analyses were performed.

## 5. Analysis

The following outcome and efficacy measures were predetermined:

1. the primary efficacy outcome was the proportion of minimal trauma fractures;
2. secondary outcomes were reported changes in bone mineral density (BMD) and bone turnover markers;
3. reported adverse events and safety outcomes.

## 6. Descriptions of Studies

There were two hundred two potentially relevant abstracts identified. After excluding studies that did not meet eligibility criteria, forty-four studies were fully reviewed for potential inclusion.

There were 13 publications [15–27] meeting the specified eligibility criteria, one of which reported extended followup of an earlier study [16]. Further 2 studies met most eligibility criteria [28, 29]. There were eight studies with discreet residential care populations [15–19, 22, 23, 28, 29], including one study [23] that was a substudy of a larger study [15]. Two studies [15, 28] included both residential and community groups and reported some outcomes separately. Where possible these groups are presented separately in this review. Six further community-based studies [20, 21, 24–27] met inclusion criteria. One study met eligibility criteria based on published subgroup analysis [21]. Where subgroup data were used a full review of the original studies was completed, although outcomes were limited to those specifically of the subgroups. Most studies included fracture outcome data. Data regarding BMD were reported in five studies [17, 18, 23, 26, 27] and biochemical data in 11 studies [15, 17, 18, 20, 22, 23, 25–29]. Three publications [23, 26, 27] measured BMD and biochemical data, but not fractures.

## 7. Descriptions of Studies in Residential Care Settings

### 7.1. Studies of Vitamin D with Calcium

Three residential studies used either cholecalciferol [17, 18] or ergocalciferol [19] with supplemental calcium. Chapuy et al. [17] reported results after 18-month treatment but subsequently reported 3-year followup [16]. The Decalyos II study [18] used two different preparations of the same overall dose of cholecalciferol and calcium (“fixed” or “split”) and differentiated between the two preparations for biochemical analysis but treated them as one group for fracture analysis. Flicker et al. [19] used ergocalciferol and calcium in a residential falls intervention and measured fractures as a secondary outcome. The placebo groups also received calcium. 

Exclusion criteria in these studies included immobility [17, 18], medications affecting bone metabolism [17–19] (although the Chapuy study allowed oestrogen and thiazide diuretics), hypercalcemia [18], chronic renal failure [18], serious health conditions [17–19], malabsorption [19], or reduced life expectancy [17, 18].

Baseline characteristics, study durations, and dose regimens are found in Table 1.

### 7.2. Studies of Vitamin D without Calcium

Five studies including residential settings used cholecalciferol [15, 23, 29] or ergocalciferol [22, 28] without calcium supplementation. There were significant community groups in two studies [15, 28]. Where possible outcomes of the residential and community groups from these studies are reported separately. Two studies were not randomized by current standards [28, 29]. Lyons et al. used 4-month oral dosing, while Heikinheimo et al. used intramuscular (IM) ergocalciferol. Meyer et al. [29] administered cholecalciferol in the form of cod liver oil, with cholecalciferol deficient cod liver oil as placebo. The study by Ooms et al. [23] measured BMD and bone turnover markers in a residential subgroup of the study by Lips et al. [15]. 

Exclusion criteria in these studies included serious illness and poor life expectancy [15, 23, 28, 29], immobility [29], hypercalcaemia [28], inability to consent or comply with medications [15, 23, 29], renal stones [15, 23], sarcoid [15, 23], past hip fracture or arthroplasty [15, 23], or preexistent vitamin D supplementation [22, 29] or general contraindications to treatment [22].

Baseline characteristics, study durations, and dose regimens are described in Table 1.

## 8. Descriptions of Community-Based Studies including Outcomes in Patients ≥70 Years of Age

### 8.1. Studies of Vitamin D with Calcium

Five community studies used cholecalciferol [20, 21, 24] or ergocalciferol [26, 27] with supplemental calcium. The RECORD trial [20] was a secondary fracture prevention trial using varying combinations of vitamin D, calcium, and placebo. Porthouse et al. [24] did not have a placebo in the control group. The Women's Health Initiative (WHI) calcium and vitamin D trial [21] contained a subgroup of 6340 women aged 70–79 years. These women were also randomized to hormone therapy if they were concomitantly enrolled in the WHI hormone trials [30, 31]. A substudy [27] of the Calcium Intake Fracture Outcome Study (Kyphos) [32] used varying combinations of calcium, ergocalciferol, and placebo. Zhu et al. [26] measured the effect of calcium with or without ergocalciferol in women with reduced vitamin D levels at baseline. 

Exclusion criteria in these studies included low life expectancy [27], risk of bone malignancy [20], poor health, hypercalcaemia [20, 21, 24], medications affecting bone metabolism [20, 21, 26, 27] (although the WHI study allowed the use of oestrogen and bisphosphonates), chronic renal failure [24, 26], inability to consent or poor cognition [20, 24, 26], renal stones [20, 24, 26], immobility [20], neurological impairment of gait [26], recent fracture [26], low BMD [26], conditions affecting bone metabolism [27], or excessive supplements of calcium [21, 24] or vitamin D [20, 21, 26]. 

Baseline characteristics, study durations, and dose regimens are described in Table 2.

### 8.2. Studies of Vitamin D without Calcium

Three community studies used cholecalciferol [15] or ergocalciferol [25, 28]. The two studies described above regarding residential subgroups [15, 28] report their findings more fully over the entire group. Smith et al. [25] used yearly IM ergocalciferol with similar entry criteria to the Heikinheimo study, but with more stringent exclusions. 

Exclusion criteria in these studies included poor health or low life expectancy [15, 25, 28], inability to give consent [15], hypercalcaemia [25, 28], chronic renal failure [25], renal stones [15, 25], sarcoidosis [15, 25], “treated osteoporosis” [25], and past hip fracture or arthroplasty [15, 25].

Baseline characteristics, study durations, and dose regimens are found in Table 2.

## 9. Fracture Risk Reduction in Residential Settings

Of the eight included studies with information provided on purely residential groups six provided fracture outcome data [15–17, 19, 22, 28, 29]. The Lips study provided subgroup analysis in residential subjects for hip fracture rates only. The data from Heikinheimo are most complete across the combined residential and community groups and as such are included later with the information on community groups.

The dose of vitamin D ranged from 400 IU daily [15, 29] to 1000 IU daily [16–19]. The average daily dose of vitamin D in the Lyons study was approximately 820 IU, although the intermittent oral dose may not have been effective throughout the dosing period [33]. Ergocalciferol was used in two studies [19, 22], while the others used cholecalciferol. Adjuvant calcium was used in 3 studies [16–19].

Total fractures were recorded in the Flicker and Lyons studies. There was a reduction in the percentage of patients sustaining fractures in the active treatment groups compared to comparator group in both studies (8% versus 11% and 12% versus 13%, resp.); however these reductions did not reach significance. The Lyons study primarily assessed fracture risk, but low recruitment reduced its statistical power, while the Flicker study was primarily a falls prevention study and did show reduced falls but may have lacked adequate power to confirm its secondary findings with regard to fractures. 

Vitamin D significantly reduced nonvertebral fractures with a relative risk reduction of 28% at 3 years in the Chapuy study (*P* < 0.01), while the other two studies recording nonvertebral fracture rates had nonsignificant reduction in fractures [18, 29]. The percentage of patients sustaining nonvertebral fracture ranged from 12% in the active treatment group of the Meyer study to 27% in the placebo group of the Chapuy study.

Hip fracture was significantly reduced in the Chapuy study (*P* < 0.02) and was nonsignificantly reduced in the Decalyos II study (*P* = 0.07). However, three other studies showed increases in hip fracture rates, although these failed to reach significance [15, 22, 29]. The percentage of patients sustaining hip fracture in the placebo groups ranged from as low as 5-6% in the Lips and Lyons studies to as high as 16% in the placebo group of the Chapuy study. 

A summary of the major fracture outcomes in the residential care setting at the latest reported time period is presented in Table 3.

## 10. Fracture Risk Reduction in Patients Aged ≥70 Years in Community Settings

Of the eight studies with community-based subjects six [15, 20, 21, 24, 25, 28] provided fracture outcome data. 

The oral dose of vitamin D was 400 IU daily in two studies [15, 21] and 800 IU daily in two further studies [20, 24]. The yearly dosing regimens of Heikinheimo and Smith are equivalent to between 411 and 822 IU daily. The limited duration of liver storage of fat-soluble vitamin D may suggest that patients in these two studies received higher per day dose for a shorter duration without supplementation for the remaining period. However doses were delivered in seasonal periods where vitamin D levels would be expected to be at their lowest. Ergocalciferol was used in two studies [25, 28], while the other studies used cholecalciferol. Three studies used supplemental calcium [20, 21, 24].

Total fractures were significantly reduced in the Heikinheimo study (*P* = 0.034). This decreased rate was only attributable to reduced fractures of the upper limb. Fractures were seen less commonly in women. The statistical effect of the residential subgroup on this finding was not reported. Two further studies showed no benefit of vitamin D on total fractures [20, 24]. The percentage of patients sustaining fractures ranged from 4% in the treatment arm of the Porthouse study to 22% in the control arm of the Heikinheimo study. 

There was no significant reduction in nonvertebral fracture seen using vitamin D in any included community study. The Smith study found an increased risk of fracture in women (age adjusted HR 1.21 95% confidence interval 1.00–1.47, *P* = 0.05). There was a nonsignificant increase in fracture rate in the Lips study. The percentage of subjects sustaining nonvertebral fracture ranged from 6% in the Smith study to 21% in the placebo arm of the mixed population of the Heikinheimo study.

Hip fracture was significantly increased in the Smith study (age adjusted HR 1.4 95% confidence interval 1.07–1.82, *P* = 0.04), with females but not males showing significant increase in risk. Hip fractures were increased in two further studies [15, 20] and were decreased in three studies [21, 24, 28], but none of these changes were statistically significant. The percentages of subjects sustaining fractures ranged from as low as 1% in the Smith study to 9% in the Heikinheimo study. 

A summary of the major fracture outcomes in the community and mixed population studies can be found in Table 4.

## 11. Bone Mineral Density Outcomes

Measurements of BMD were eligible for inclusion in five studies [17, 18, 21, 23, 26, 27]. The number of patients receiving BMD measurements within each study and the length of followup both varied. Values were generally reported as percentage gain in BMD versus comparator group. 

### 11.1. Studies of Vitamin D with Calcium

Bone mineral density at the femoral neck, total proximal femur, trochanter, and total hip was significantly higher compared to placebo in the Chapuy study. The Decalyos II study showed reductions in rate of both the annualised and overall bone losses, but these findings did not reach significance (*P* = 0.09).

One community-based study [27] showed decreased bone loss at the hip compared to control, but the regimen of ergocalciferol combined with calcium was not clearly superior to calcium alone in this setting [26]. No other significant BMD changes were found at any site in community-based studies.

There was no published evidence for benefit of vitamin D on total body and intertrochanteric or distal radius BMD. Spinal BMD was not reported in any studies that met eligibility criteria. 

### 11.2. Studies of Vitamin D without Calcium

The Ooms study of lower dose cholecalciferol supplementation (400 IU) reported a significant BMD benefit at the femoral neck but not other sites studied.

The results of BMD investigations of the eligible studies are shown in Table 5.

## 12. Markers of Bone Turnover

Where bone turnover markers were measured sample sizes were generally smaller than the overall size of the studies. The Heikinheimo study reported community and residential group biochemical findings separately. The Lips study measured vitamin D in 96 patients who were not included in the Ooms study.

Eleven studies measured vitamin D [15, 17, 18, 20, 22, 23, 25–29]. Baseline 25-hydroxyvitamin D (25(OH)D) was generally less than 50 nmol/L but was above 50 nmol/L in the Kyphos substudy and was 141 ± 59 nmol/L in the biochemical samples from the Smith study. Vitamin D levels were raised in the intervention arms of all eleven studies. After treatment 25(OH)D was above 50 nmol/L in 10 studies [15, 17, 18, 20, 22, 23, 25–27, 29] and was above 75 nmol/L in four studies [17, 22, 25, 27].

There was a significant reduction of parathyroid hormone (PTH) in the treatment groups of both the Chapuy study and the Decalyos II study. The PTH was significantly lower compared to placebo in two further residential studies [22, 23]. Conversely PTH was nonsignificantly raised in one study [29]. The Kyphos substudy showed significantly lower PTH levels compared to control only if baseline PTH was above the median. Three community-based studies showed nonsignificantly lower PTH in treatment groups [20, 25, 26]. The RECORD trial reported lower PTH in both treatment and placebo groups. An annual injection of ergocalciferol initially lowered PTH, but this increased towards baseline by eight months [25].

The level of alkaline phosphatase (ALP) rose significantly from baseline in the placebo but not treatment groups of the Chapuy study. While ALP decreased after the second injection in the Heikinheimo study, the magnitude of this decrease is not reported. There was significant reduction of ALP compared to control in the Kyphos study. Bone-specific ALP was significantly reduced in the treatment group of the Decalyos II study, but not the Ooms study.

There was evidence for lower serum procollagen type I intact N-terminal propeptide (P1NP) in the Zhu study compared to baseline (*P* < 0.05) but no difference compared to the calcium alone arm. Urinary deoxypyridinoline to creatinine ratios were significantly reduced compared to both placebo and calcium in the Kyphos study at 5 years (*P* = 0.002 and *P* = 0.03, resp.). There was no evidence for changes in osteocalcin [17, 23, 29] or urinary hydroxyproline/creatinine ratios [23].

The major biochemical findings of the included studies at the last reported followup are shown in Table 6. Where authors did not report absolute values we have presented *P* values only.

## 13. Adverse Effects Reported

Vitamin D containing regimens were well tolerated in the included studies. While the reporting of adverse events varied between trials, the rate of adverse events appears low. Several studies reported gastrointestinal side effects [17, 18, 20, 21]. The RECORD trial found that gastrointestinal side effects related to calcium treatment rather than vitamin D. Hypercalcaemia was rare in all trials. Despite some evidence for increases in urinary calcium-creatinine ratios [18, 23], there was no clear increase in renal stones or renal impairment. There were no significant differences in mortality reported [15, 17–19, 21, 22, 28, 29], nor were there any recorded significant differences in rates of malignancy [21, 26, 28] or cardiovascular disease [26–28], although these outcomes were not reported frequently.

## 14. Discussion

Despite meta-analyses [34–37] suggesting benefit in treating the elderly and residential care patients with vitamin D, the evidence for this is limited to specific situations. There is heterogeneity of studies of vitamin D to treat osteoporosis, and findings from these meta-analyses may not be applicable to all the elderly [38], Positive results from trials with younger populations [39, 40] have been included in past meta-analyses, but these may not reflect the residential populations or the community groups included in this review. 

In both residential and community studies there was heterogeneity of treatment, with both cholecalciferol and ergocalciferol having varying doses, routes of administration, and dose frequency. The studies are further complicated by the presence or absence of calcium supplementation in both the residential care and community populations. The lack of consistency in dose limits the ability to draw direct comparison between community and residential groups.

Improved fracture outcomes in the residential care setting have only been demonstrated by combining calcium and cholecalciferol at a dose of 800 IU daily in patients who have poor calcium intake and are vitamin D deficient [16, 17]. None of the studies using ergocalciferol with or without calcium demonstrated significant fracture reduction. Fracture rates varied between residential studies, which may reflect the country the study took place in, varying diet, comorbidity within study populations, falls prevention strategies [41], or other factors that may alter the background rate of fracture in residential settings, thus potentially reducing the ability of studies to detect a true benefit of vitamin D treatment. 

There is no clear evidence for fracture reduction in community patients aged ≥70 with either ergocalciferol or cholecalciferol. The only studies showing any difference were the Heikinheimo study, which showed limited fracture reduction, and the Smith study, which demonstrated an increase in hip fracture by 49% in the treatment group. This increase in hip fracture is difficult to explain but could possibly relate to excessive calcium absorption, given both that this was a population likely to be vitamin D replete at baseline and that calcium supplementation has recently been suggested to possibly increase hip fracture risk [42]. The Heikinheimo and Smith studies used yearly IM ergocalciferol but are clearly conflicting. Given the incomplete nature of the Heikinheimo study data and the increased hip fracture risk in the Smith study, yearly IM ergocalciferol cannot be recommended for fracture prevention. 

The only secondary prevention trial of calcium and vitamin D was the RECORD trial, which was negative. Vitamin D without calcium cannot currently be recommended as a sole treatment regimen for secondary prevention of fracture, although a study of predominantly vitamin D deficient community dwelling individuals has shown improvement of BMD and falls in the secondary prevention setting [43].

Since the completion of this review a further community based trial has been reported. This double-blind trial enrolled 2256 women aged 70 years or older and randomized them to either 500000 IU choleciferol for placebo annually for 3 to 5 years [44]. The relative risk for fracture in the vitamin D group was higher at 1.26 (CI—1.02–1.30; *P* = 0.03) with a rate per 100 person-years of 4.9 versus 3.9. This may have been attributable to the higher rate of falls also shown in the vitamin D group (83.4 per 100 person-years versus 72.7 person-years). Most falls occurred in the initial 3 months of the intervention period versus later (1.31 versus 1.13). The exact reason for this is unclear. One hypothesis may be that high dose vitamin D supplementation increases mobility, but in an unsafe manner with an increased falls risk as a consequence. The suggestion has also been made that high-dose vitamin D may paradoxically increase 1,25(OH)_2_-vitamin D catabolism by increasing the action of CYP24 hydroxylase [45]; however the Smith study [25] reviewed here showed an increase in 1,25(OH)_2_-vitamin D, as did a subsequent study of high-dose ergocalciferol [45], which would appear to make this theory less likely. It has been noted that fibroblast growth factor-23 (FGF-23), which may also stimulate CYP24 hydroxylase so as to avoid hypercalcaemia, is increased in response to a high dose of ergocalciferol. An increase in FGF-23 may also contribute to poor bone health via other possible pathways including suppression of osteoblasts and poor mineralisation [45]. A high loading dose of cholecacilferol (600,000 IU) was also recently found [46] to acutely increase C-terminal-telopeptides of type I collagen and cross-linked N-telopeptide of type I collagen. While previous studies have not shown these changes, such an acute increase in markers of bone resorption may also help to explain the early increase risk of fractures seen in the recent community trial [44].

The mechanism for any fracture reduction with vitamin D appears to be reduction in secondary hyperparathyroidism induced by hypovitaminosis D and resolution of osteomalacia rather than by improving the underlying age-related causes of osteoporosis in older people. The included studies showed no improvement in markers of bone formation, with no evidence of anabolic action with these vitamin D preparations and doses, despite the presumed potential of vitamin D to have an anabolic effect via osteoblastic activity. Levels of ALP were either lowered from baseline or did not rise compared to placebo groups, again more consistent with reversal of hyperparathyroidism in treatment groups and osteomalacia in comparator groups. Fracture reduction in the Chapuy trial was associated with an increase in 25(OH)D to 105 nmol/L. The only other study to demonstrate a 25(OH)D increase to comparable levels was the Kyphos substudy, which showed BMD and biochemical improvements with ergocalciferol. These findings and levels of 25(OH)D attained in positive studies that did not meet inclusion criteria [39, 40] concur with recommendations for serum 25(OH)D to be maintained at a level above 75 nmol/L [47]. Although biochemical “normalisation” of PTH was demonstrated at vitamin D levels of 50 nmol/L, it may be that this vitamin D level and effect on PTH are insufficient to obtain fracture benefit, despite some evidence for BMD improvements. Hence the concept of “optimisation” of vitamin D and PTH may be more important for bone health. This may need to be more clearly investigated and defined.

While evidence exists for decreased body sway and reduction in falls with vitamin D supplementation, particularly in vitamin D deficient settings [19, 48–52], this did not translate into a statistically significant reduction in fracture in the Flicker study despite reduction in falls. This study was not powered to obtain a statistical result. 

Poor compliance or infrequent dosing regimens may explain some of the lack of effect seen in community studies, particularly if a level of 75 nmol/L is desirable. Certainly the only effect in the overall WHI trial was seen in those subjects who were compliant with medication use [21]. The lack of effect in community groups may suggest “contamination” in placebo or control arms of the studies and might explain the mild drop in PTH noted in the placebo group of the RECORD study. However, community groups, apart from the Zhu study, generally appeared to have vitamin D levels near “normal” at baseline, which may have negated the need for any further vitamin D. As such it appears more likely that vitamin D supplementation has no beneficial role on bone health in a replete population, particularly if calcium intake is adequate. Community studies generally had more stringent exclusion criteria, which may have biased the studies towards healthier subjects without secondary causes of osteoporosis or the tendency to hypovitaminosis D. These studies are therefore likely to have excluded the frail community living individuals at high risk for fracture and who may be more likely to benefit from treatment. 

The type of vitamin D used and dosing regimen varied, and this may explain variations in outcomes between studies. Cholecalciferol has been shown to be superior to ergocalciferol at raising 25(OH)D levels [53, 54] and appears to be the preparation of choice based on the available evidence. However, a difference between cholecalciferol and ergocalciferol may not be clinically relevant [55], and the benefit in BMD and biochemistry in the Kyphos substudy and improvement in falls by ergocalciferol in vitamin D deficient populations [19, 51] may suggest that the two forms of vitamin D preparation are comparable. Yearly intramuscular dosing may not provide sufficient PTH suppression throughout the year, as evidenced by the small biochemical sample in the Smith trial. Daily dosing may be more effective than infrequent dosing [33], but recent evidence suggests that monthly dosing may also be effective at raising 25(OH)D levels [56].

Very few studies reported BMD outcomes. Significant improvement in femoral neck BMD has been demonstrated for 800 IU of cholecalciferol with calcium (although this effect was not seen in the Decalyos II trial) and 400 IU of cholecalciferol as a single agent. The only benefit seen in the community setting was improvement in total hip BMD after 5 years of treatment with ergocalciferol in the Kyphos substudy. The improvement in BMD in areas of cortical bone does suggest some benefit of vitamin D in patients with osteoporosis associated with aging, although the BMD finding did not translate into fracture prevention. Although BMD is a strong risk factor for fragility fracture, its relative importance is attenuated with age, which may explain the lack of discrepancy between BMD and fracture rates seen here [57].

Treatment of the community dwelling elderly with regimens including calcium may need to be individualised based on fracture risk and vitamin D status given recent concern regarding cardiovascular risk associated with calcium supplementation in healthy elderly women [58]. Varying levels of calcium intake [59] and reduced absorption in the vitamin D deficient elderly [12] makes it hard to generalise this finding to other populations. It is reassuring that calcium in combination with vitamin D did not have any cardiovascular effects noted in the studies included in this review, although this outcome was not commonly recorded. Improving vitamin D may have a role in improving cardiovascular illness [12] and could attenuate any risk associated with calcium supplementation. The duration of followup in the included trials may not be sufficient to detect other long-term health risks; however it is reassuring that evidence of reduced cancer risk exists in a younger population [60].

## 15. Conclusions

Despite the heterogeneity of the evidence presented here, it appears that the greatest benefit of vitamin D on fractures and BMD occurs with cholecalciferol with additional calcium in frailer residential care settings. The mechanism appears most likely to be due to resolution of secondary hyperparathyroidism and osteomalacia, which may explain the negative results in vitamin D replete community dwelling individuals. Frail, vitamin D deficient individuals living in the community may best approximate residential care patients [61] and would appear to be the most likely to benefit from vitamin D and calcium treatment. Current medications used for fracture prevention in the community may not be effective in the elderly [62], and anabolic agents may need to be considered to help prevent osteoporosis in this population [11, 62]. Recent cardiovascular findings associated with calcium challenge assumptions of safety in vitamin D and calcium containing regimens. The goal should be to optimise calcium intake to meet recommended daily requirements through diet and supplementation when diet is inadequate. This may minimise any potential cardiovascular concerns and risks identified in studies suggesting harm [58]. Research into primary and secondary fracture prevention with vitamin D in the frail community dwelling elderly is required, both to identify safe fracture prevention strategies and to redress the lack of evidence in those most likely to benefit from interventions reducing osteoporotic fracture.

## Figures and Tables

**Table 1:** Characteristics of residential studies.

	Duration	Treatment	Dose (IU)	Regimen	Number	Female (%)	Age mean (SD)	Vitamin D (nmol/L)^# ^mean (SD) or median (IQR)	Ca+ intake (mg/day) mean (SD) or median (IQR)
Chapuy	3 years	All			3270	100	84 (6)	NA	513
		D3	800 daily	CaD	1634		84 (6)	40 (28)	511 (172)
		P		P	1636		84 (6)	33 (23)	514 (158)
Decalyos II	2 years	All			583	100	85 (7)		557.7
		D3	800 daily	CaD fixed CaD split	393		85 (7) 85 (7)	21 (13) 23 (17)	565 (230) 551 (238)
		P		P	190		86 (8)	23 (17)	556 (246)
Flicker	2 years	All			625	95	83	25–90	NA
		D2	10000 weekly^§^	CaD	312		84 (8)	NA	NA
		P		CaP	313		83 (9)	NA	NA
Lips-combined	Max. 3.5 years	All			2578	74	80 (6)		NA
		D3	400 daily	D	1291		80 (6)	27 [19–36]	NA
		P		P	1287		80 (6)	26 [19–37]	NA
Ooms	2 years	All			348	100			
		D3	400 daily	D	177		80 (6)	68 [48–90]	876 [638–1101]
		P		P	171		81 (6)	63 [48–93]	859 [644–1099]
Meyer	2 years	All			1144	75	85 (7)		
		D3	440 daily	D	569		84 (7)	47 (26)	456 (196)
		P		P	575		85 (7)	51 (33)	446 (196)
Heikinheimo residential	Range 2–5 years	All			320	82	NA	NA	NA
		D2	150000–300000 yearly*	D	142		79	NA	NA
		P		P	178		79	NA	NA
Lyons	3 years	All			3440	76	84 (8)	NA	NA
		D2	100000 4 monthly	D	1725	76	84 (8)	NA	NA
		P		P	1715	77	84 (8)	NA	NA

*IM treatment.

^§^Changed to 1000 IU daily during the course of the study.

^#^Divide by 2.5 for ng/mL. IU: international units; D2: ergocalciferol; D3: cholecalciferol; D: vitamin D; CaD: calcium and vitamin D; Ca: calcium; P: placebo or control; NA: not available.

**Table 2:** Characteristics of community studies.

	Duration	Treatment	Dose (IU)	Regimen	Subjects	Female (%)	Age mean (SD) or median (range)	Vitamin D (nmol/L)^# ^mean (SD) or median (IQR)	Ca+ intake (mg/day) mean (SD) or median (IQR)
RECORD	Median 45 months (range 24–62)	All			5292	85	77 (6)	NA	NA
		D3	800 daily	D/CaD	2649		77 (6)	NA	NA
		P		Ca/P	2643		77 (6)	NA	NA
Porthouse	Median 25 months (range 18–42)	All			3314	100		NA	
		D3	800 daily	CaD	1321		77 (5)	NA	1075 (338)
		P		P	1993		77 (5)	NA	1084 (346)
WHI subgroup	7 years (mean)	All			6340	100	>70	NA	1150
		D3	400 daily	CaD	3173			NA	NA
		P		P	3167			NA	NA
Kyphos substudy	1 year	All			120	100	75 (3)	68 (29)	1010 (349)
		D2	1000 daily	CaD	39		75 (3)	70 (26)	927 (295)
		P		P CaP	41 40		75 (3) 74 (2)	67 (34) 67 (26)	1046 (340) 1054 (398)
Zhu	5 years	All			302	100		44 (13)	1097 (495)
		D2	1000 daily	CaD	151		77 (4)	45 (13)	1109 (503)
		P		CaP	151		77 (5)	44 (13)	1087 (410)
Lips-combined	3.5 years maximum	All			2578	74	80 (6)		NA
		D3	400 daily	D	1291		80 (6)	27 [19–36]	NA
		P		P	1287		80 (6)	26 [19–37]	NA
Heikinheimo community	2–5 years	All			479	77		NA	NA
		D2	150000–300000 yearly*	D	199		87	NA	NA
		P		P	280		86	NA	NA
Smith	3 years	All			9440	54	79 [77–83]	141 (59)	625 (231)
		D2	300000 yearly*	D	4727		79 [77–83]	NA	NA
		P		P	4713		79 [77–83]	NA	NA

*IM treatment. ^#^Divide by 2.5 for ng/mL. IU: international units; D2: ergocalciferol; D3: cholecalciferol; D: vitamin D only; CaD: calcium and vitamin D; Ca: calcium only; P: placebo or control; NA: not available.

**Table 3:** Fracture outcomes in residential care settings.

	Duration	Regimen	Compliance (%)	Total fracture patients (%)	Non-vertebral fracture patients (%)	Hip fracture patients (%)
Chapuy	3 years	CaD	83	NA	255 (22)	137 (12)
		P	84	NA	308 (27)	178 (16)
		RRR		NA	28% (*P* < 0.01)	27% (*P* < 0.02)
Decalyos II	2 years	CaD	95	NA	17.8%	27 (6.9)
		P		NA	17.9%	21 (11.1)
		RRR		NA	NS	NS (*P* = 0.07)
Flicker	2 years	CaD	95	25 (8)	NA	NA
		CaP		35 (11)	NA	NA
		RRR		NS	NA	NA
Lips subgroup	Max. 3.5 years	D	85	NA	NA	49 (6)
		P		NA	NA	36 (5)
		RRR		NA	NA	NS
Meyer	2 years	D	79%	NA	69 (12)	50 (9)
		P		NA	76 (13)	47 (8)
		RRR		NA	NS	NS
Lyons	3 years	D	85	205 (12)	NA	112 (6)
		P		218 (13)	NA	104 (6)
		RRR		NS	NA	NS

ITT analysis unless otherwise stated. D: vitamin D; CaD: calcium and vitamin D combination treatment; P: placebo or control; NA: not available; NS: not significant; RRR: relative risk reduction.

**Table 4:** Fracture outcomes in community and mixed population studies.

	Duration	Regimen	Compliance (%)	Total fracture patients (%)	Non-vertebral fracture patients (%)	Hip fracture patients (%)
RECORD	Median 45 months (range 24–62)	D/CaD	47–60	353 (13)	349 (13)	93 (4)
		CaP/P		345 (13)	341 (12)	90 (3)
		RRR		NS	NS	NS
Porthouse	Median 25 months (range 18–42)	CaD	63	58 (4)	NA	8 (1)
		P		91 (5)	NA	17 (1)
		RRR		NS	NA	NS
WHI subgroup	7 years (mean)	CaD	59	NA	NA	115 (3)
		P		NA	NA	93 (4)
		RRR		NA	NA	NS
Heikinheimo	Range 2–5 years	D	NA	56 (16)	48 (14)	25 (7)
		P		100 (22)	94 (21)	43 (9)
		RRR		NR (*P* = 0.034)	NA	NS
Lips	Maximum 3.5 years	D	85	NA	135 (10)	58 (4)
		P		NA	122 (9)	48 (4)
		RRR		NA	NS	NS
Smith	3 years	D	NA	NA	306 (6)	66 (1)
		P		NA	279 (6)	44 (1)
		RR Increase		NA	NS	49% (*P* = 0.04)

ITT analysis unless otherwise stated. D: vitamin D; CaD: calcium and vitamin D combination treatment; P: placebo or control; RRR: relative risk reduction; NA: not available; NS: not significant; RRR: relative risk reduction; NS: not significant; NR: not reported; NA: not available.

**Table 5:** Results of community and residential studies measuring BMD.

	Sample size	Regimen	Maximum followup	Sites	BMD change versus baseline (%)		
					Active	Comparator	*P *
Chapuy	56	CaD versus P	18 months	Femoral neck Total proximal femur Trochanter Intertrochanteric	+2.9 ± 6.4 +2.7 ± 5.5 −1.0 ± 6.9 +1.1 ± 4.7	+1.8 ± 9.4 −4.6 ± 9.2 −6.4 ± 12.3 +3.2 ± 20.1	0.036 <0.001 0.044 NS
Decalyos II	114	CaD versus P	2 years	Femoral neck Distal radius*	−1.2 ± 7.4	−4.5 ± 7.1	NS
	583				NA	NA	NS
Zhu	302	CaD versus CaP	1 year	Total body Total hip	+0.4 ± 0.2 +0.5 ± 0.3	+0.4 ± 0.2 +0.2 ± 0.2	NS NS
Kyphos	120	CaD versus P	5 years	Total hip	−0.4 ± 0.7	−2.5 ± 0.6	0.05
Ooms	348	D versus P	2 years	L femoral neck R femoral neck L femoral trochanter R femoral trochanter Distal radius	+1.6 +1.2 −1.1 −0.9 −2.7	−0.3 −1.4 −0.9 −0.9 −2.4	0.01 0.001 NS NS NS

Values are mean ± standard deviation; NS: not significant; CaD: calcium and vitamin D; P: placebo or control; NA: not available. *Single X-ray absorptiometry.

**Table 6:** Effect of vitamin D on bone turnover markers.

	Sample size	Time	Regimen	25(OH)D (nmol/L^¶^)		PTH (pmol/L)		ALP (U/L)	
				Baseline	Followup	Baseline	Followup	Baseline	Followup
Chapuy	142	18 months	CaD P	40 ± 28 33 ± 23	105 ± 23* 28 ± 18	5.9 ± 4.1 5.5 ± 2.6	3.3 ± 1.5* 6.2 ± 3.2^§^	69 ± 25 72 ± 25	67 ± 22 89 ± 27^§^

Decalyos II	583	2 years	CaD fixed CaD split P	21 ± 13 23 ± 17 23 ± 17	↑^#^ ↑^#^ NR	7.8 ± 8.4 7.8 ± 5.6 7.9 ± 5.3	↓^#^ ↓^#^ ↑	NA NA NA	NA NA NA

RECORD	60	1 year	CaD D P	Combined 38 ± 16	+24 ± 17 +24 ± 22 +8 ± 18	Combined 5.5 ± 2.4	−1.9 ± 2.2 −0.7 ± 1.6 −0.7 ± 1.5	NA NA NA	NA NA NA

Zhu	302	1 year	CaD Ca	45 ± 13 44 ± 13	+34%* NA	7.8 ± 3.0 7.9 ± 3.1	−3.7 ± 2.6% −2.8 ± 2.5%	NA NA	NA NA

Kyphos sub-study	120	5 years	CaD Ca P	70 ± 26 67 ± 26 67 ± 34	106 ± 29^#§^ 64 ± 29 62 ± 34	3.7 ± 1.3 3.9 ± 1.7 3.9 ± 1.2	NA NA NA	80 ± 21 84 ± 18 83 ± 18	↓^‡^ NA NA

Heikinheimo residential	33	Final treatment + 1 year	D P	NA NA	45 ± 16 14 ± 5	NA NA	NA NA	NR NR	NR NR

Heikinheimo community	29	Final treatment + 1 year	D P	NA NA	49 ± 14 31 ± 14	NA NA	NA NA	NR NR	NR NR

Lips	96	3 years	D P	NA NA	54 (43–61)^#^ 23 (17–28)	NA NA	NA NA	NA NA	NA NA

Ooms	270	1 year	D P	27 (19–36) 26 (19–37)	62 (52–70)^#^ 23 (17–31)	3.3 (2.4–4.6) 3.5 (2.7–5.0)	3.1 (2.3–4.1)^‡^ 3.7 (2.5–5.8)	63 (53–78) 66 (55–78)	62 (53–74) 66 (54–76)

Meyer	70	1 year	D P	47 ± 26 51 ± 33	64 ± 21^#^ 46 ± 20	6.5 ± 3.1 5.6 ± 3.3	7.5 ± 4.2 7.2 ± 4.7	NA NA	NA NA

Lyons	102	At least 5 doses	D P	NA NA	80.1^*δ*^ 54.0	NA NA	5.0^*γ*^ 6.65	NA NA	NA NA

Smith	43	4/12	D P	Combined 141 ± 59	+21% NA	*4.8 (3.9–5.6) *	−17% NA	NA NA	NA NA

Ca: calcium; D: vitamin D; P: placebo; NA: not available; NR: not recorded; ↓: decreased; ↑: increased.

Plus-minus values: means ± SD. Values followed parentheses: median (interquartile range). Results nonsignificant unless indicated.  ^¶^To convert to ng/mL divide by 2.5.  *P ≤ 0.005 compared to baseline.  ^§^ *P* ≤ 0.05 compared to baseline.  ^#^ *P* ≤ 0.005 compared to placebo.  ^‡^ *P* ≤ 0.05 compared to placebo.  ^*δ*^Difference between means 10.4 ng/mL (95% CI: 6.6–14.2).  ^*γ*^Difference between means 1.65 pmol/L (95% CI: 0.54–2.74).
